# Ambidextrie ist Ambidextrie ist Ambidextrie …

**DOI:** 10.1365/s40702-023-00985-x

**Published:** 2023-06-05

**Authors:** Sara D’Onofrio, Carolin Durst, Susanne Robra-Bissantz

**Affiliations:** 1 IT Business Integration, Genossenschaft Migros Zürich, Zürich, Schweiz; 2 Fakultät Wirtschaft, Hochschule für angewandte Wissenschaften Ansbach, Ansbach, Deutschland; 3 Institut für Wirtschaftsinformatik, Technische Universität Braunschweig, Braunschweig, Deutschland

**Keywords:** Ambidextrie, Digitalisierung, Exploit, Explore, Führung, Innovationsmanagement, Kultur, Ambidextry, Culture, Digitalization, Exploit, Explore, Leadership, Innovation Management

## Abstract

Ambidextrie im Innovationsmanagement bezieht sich auf die Fähigkeit eines Unternehmens, sowohl das Kerngeschäft zu stärken (*Exploit*) als auch mit neuen Angeboten in bestehenden oder neuen Märkten zu agieren (*Explore*). Dabei besteht die Herausforderung darin, beide Innovationsmodi in einer komplementären Art und Weise einzusetzen, anstatt sich auf einen der beiden Modi zu beschränken. Eine ambidextre Ausrichtung und die Fähigkeit, beide Innovationsmodi, Exploit und Explore, gleichzeitig und in dem Maß einzusetzen, das die Umstände erfordern, ermöglicht es Unternehmen, schnell auf Veränderungen in der Branche zu reagieren und sich langfristig im Markt zu behaupten. Für eine erfolgreiche Umsetzung von Ambidextrie im Innovationsmanagement spielen die Unternehmenskultur, der Führungsstil resp. das Führungsverständnis ebenso eine Rolle, wie Grundsatzentscheidungen in Bezug auf die Digitalisierung. Es ist entscheidend, dass Unternehmen die Potenziale von Ambidextrie erkennen und lernen, den Exploit- und Explore-Modus gezielt und optimal einzusetzen, um langfristig Wettbewerbsvorteile zu erzielen. Dieser Grundlagenartikel fokussiert insbesondere auf die im weiteren Heft weniger angesprochenen Themenbereiche. Er ordnet die Ambidextrie im Innovationsmanagement anhand der Themenfelder Kultur, Führung und Digitalisierung ein und liefert Argumente, weshalb die Akzeptanz und Nutzung von Ambidextrie zu Wettbewerbsvorteilen führen.

## Abstract (en)

Ambidexterity in innovation management refers to a company’s ability to both strengthen its core business (*exploit*), as well as to act with new offerings in existing or new markets (*explore*). The challenge is to use both innovation modes in a complementary way, rather than limiting oneself to one of the two modes. An ambidextrous approach and the ability to use both innovation modes, exploit and explore, simultaneously and to the extent required by the circumstances, enables companies to quickly respond to changes in the industry and thus succeed in the market in the long term. To successfully implement ambidexterity in innovation management, corporate culture and leadership style play just as important a role as fundamental decisions regarding digitization. It is crucial for companies to recognize the potentials of ambidexterity and learn to use both the exploit and explore modes specifically and optimally to achieve long-term competitive advantages. This foundational article focuses in particular on the topics less addressed in the rest of the issue. It contextualizes ambidexterity in innovation management in terms of culture, leadership, and digitization, and provides arguments for why the acceptance and use of ambidexterity lead to competitive advantages.

## Ambidextrie – Ein Begriff im Wandel

„*Eine Rose ist eine Rose ist eine Rose*“. Eine Rose ist nun einmal einfach eine Rose und sonst nichts. Hat Gertrude Stein das gemeint? Oder meinte sie viel mehr, dass die Rose ganz unterschiedliche Konnotationen, Gefühle, innere Bilder und Einstellungen mit sich bringen kann: als etwas Schönes, als etwas Stacheliges und Schmerzhaftes oder als feiner und luxuriöser Duft? Das Zitat kann auch bedeuten, dass das erste Erscheinen von Rosen in Kunst und Kultur tatsächlich die Blume bezeichnete. In späteren Werken und mit zunehmender Bekanntheit jedoch wandelte sich das Verständnis hin zu Rosen als Symbol für Schönheit, Liebe, Stolz oder Leiden.

Von Rosen zur Ambidextrie: Schlägt man den Begriff das erste Mal in einem Lexikon nach oder gibt ihn in eine Suchmaschine ein, so erscheint genau das, was Ambidextrie nun einmal ist: *Beidhändigkeit* – abgeleitet aus dem lateinischen *ambi* für „beide“ und *dextra* u. a. für „richtig, geschickt“. Wie im Fall der Rose entstehen Konnotationen. Zunächst das Bild eines Menschen, der die besondere Fähigkeit besitzt, beide Hände gleich geschickt einzusetzen. Sofort ergänzt sich dieses Bild durch die Erfahrung, dass dies schwierig zu erlernen ist: man erinnert sich daran, dass in früheren Zeiten versucht wurde, linkshändige Erstklässlerinnen und Erstklässler auf den Einsatz der rechten Hand umzuerziehen – und dass dieser Versuch im modernen Unterricht nicht mehr unternommen wird. Ambidextrie steht damit für etwas ganz Besonderes – aber auch für etwas so sehr schwer zu Erlernendes, dass es kaum gelingen kann.

Wie im Fall der Rose wandeln sich auch die Konnotationen der Ambidextrie. So bezieht sie sich heute nur noch metaphorisch auf die Nutzung beider Hände, und damit eher auf die Flexibilität sowie vielfältige Gaben oder Kompetenzen, die auf den ersten Blick gegensätzlich erscheinen. Ambidextrie steht zunehmend für eine Fähigkeit, die dringend zu erwerben ist, um für Lebenswelten, die von stetem Wandel, von Unsicherheiten und Herausforderungen geprägt sind, gewappnet zu sein.

Da ist bereits im Privaten das berufstätige Elternteil, das im steten Wechsel oder auch gleichzeitig völlig unterschiedlich „privat liebevoll“ oder „geschäftlich präzise“ handeln soll und häufig mit mehr als zwei Händen alle Aufgaben jongliert. Auch in Unternehmen kann und muss Ambidextrie gelernt werden (z. B. Duwe 2018). Bei näherer Betrachtung hat sich dabei die Konnotation der beiden früher für alle Aufgaben gleichberechtigt zu nutzenden Hände der Ambidextrie vielleicht nicht geändert, aber zumindest im Unternehmensumfeld geschärft. Die zweite Hand soll dazu genommen und befähigt werden. Jedoch sollen die beiden Hände unterschiedliche Aufgaben erfüllen: Aufgrund des stetigen Anpassungsdrucks der wohl strukturierten Geschäftstätigkeit an eine sich wandelnde, unsichere und veränderliche Umwelt, steht eine Hand für wohl bekannte, bereits gelernte, eingeführte Prozesse und die andere für das, was man noch nicht kann, jedoch flexibel und divergent angehen und neu denken sollte.

Das umfasst in einem organisationalen Lernen, Ambidextrie zu kennen und zu verstehen. Erschwerend kommt hinzu, dass neues Lernen gerade in der Ambidextrie auch *verlernen* bedeutet. Dabei ist es für das traditionelle Unternehmen nicht leicht, seine seit langem eingeführten Strukturen und Prozesse mit modernen, an das heutige Arbeitsumfeld angepassten Führungsformen anzureichern oder sogar durch diese zu ersetzen. Ebenso schwer fällt es jedoch beispielsweise dem kreativen und im kleinen Team auf Zuruf und flexibel arbeitenden Start-up, seine Arbeitsweise daran anzupassen, dass es unter Umständen schnell viele Mitarbeitende aufnimmt und in diesem neuen Markt stabil und prozessorientiert agieren muss.

Natürlich reicht *verstehen *nicht. Lernen bedeutet auch, Verstandenes anzuwenden, zu analysieren, zu evaluieren, strategisch zu reflektieren und sich oder das Gelernte eigenständig im eigenen Umfeld weiterzuentwickeln. Für die Anwendung des konzeptuellen Wissens zur Ambidextrie sind Fähigkeiten und damit prozedurales Wissen notwendig. Welche Methoden können die gewohnte Vorgehensweise durch die neue ergänzen? Welche strukturellen Veränderungen der Gesamtorganisation eröffnen Raum für das neue Handeln? Wie ergänzt man bisherige Strategien durch die neuen? Spätestens wenn es im Unternehmen um die strategische oder eine persönliche Weiterentwicklung in Richtung Ambidextrie geht, werden Fragen dazu laut, wie sich, neben der Strategie selbst, auch die Menschen im gesamten Unternehmen, mit ihrem Führungsstil oder der Ausrichtung an einer gemeinsamen Kultur verändern und dies auch schrittweise lernen können. Damit steht die Frage im Raum, was es für eine Unternehmensleitung bedeutet, Ambidextrie nicht nur zuzulassen, sondern strategisch und kulturell zu verankern und zu leben – um sie damit langfristig als Leitlinie für alle Mitarbeitende zu etablieren.

In diesem Beitrag betrachten wir, für das Innovationsmanagement, insbesondere die letzteren Aspekte: Führungshandeln und Kultur, die das organisationale Lernen von Ambidextrie und damit die Ergänzung von Bekanntem durch das neue Handeln fördern. Dies rundet, so unsere Meinung, die weiteren Artikel dieser Ausgabe ab, die vor allem wesentliche Beiträge für die gezielte Anwendung, Umsetzung und Evaluation stärker ambidexter Organisationsformen, Strategien oder Methoden im Innovationsmanagement aufzeigen. Wir schließen den Beitrag mit Aspekten der Wirtschaftsinformatik ab und zeigen, wie die Digitalisierung einen wesentlichen Beitrag zum Aufbau von Wettbewerbsvorteilen durch Ambidextrie leisten kann.

## Ambidextrie im Innovationsmanagement

Ambidextrie im Innovationsmanagement fokussiert die unternehmerische Innovationstätigkeit. Grundsätzlich ist jedes Unternehmen gezwungen zu innovieren, denn kein Produkt und keine Dienstleistung werden für ewig bestehen. Dies kann unterschiedliche Gründe haben: neue Technologien entwickeln sich, Kundenbedürfnisse verändern sich, neue Wettbewerber drängen in den eigenen Markt.

Unternehmen reagieren entsprechend unterschiedlich. Sie arbeiten, aus dem Blickwinkel von Technologie-Lebenszyklen, an Innovationen, die auf evolutionären oder auf disruptiven Technologien basieren. Oder sie schlagen unterschiedliche Diversifikationsrichtungen ein und fokussieren Produkt‑, Markt- oder laterale Innovationen. Sie innovieren damit eher inkrementell oder in größeren Schritten bis dahin, dass sie eine sogenannte *Sprunginnovation* anstreben, die bedeutende soziale, ökologische oder technische Probleme löst, indem sie marktliche, technische, aber auch gesellschaftliche Aspekte zusammenführt (auch: *soziale Innovation*), völlig neue Märkte schafft oder bestehende Märkte grundlegend verändert.

**Abb. 1:**
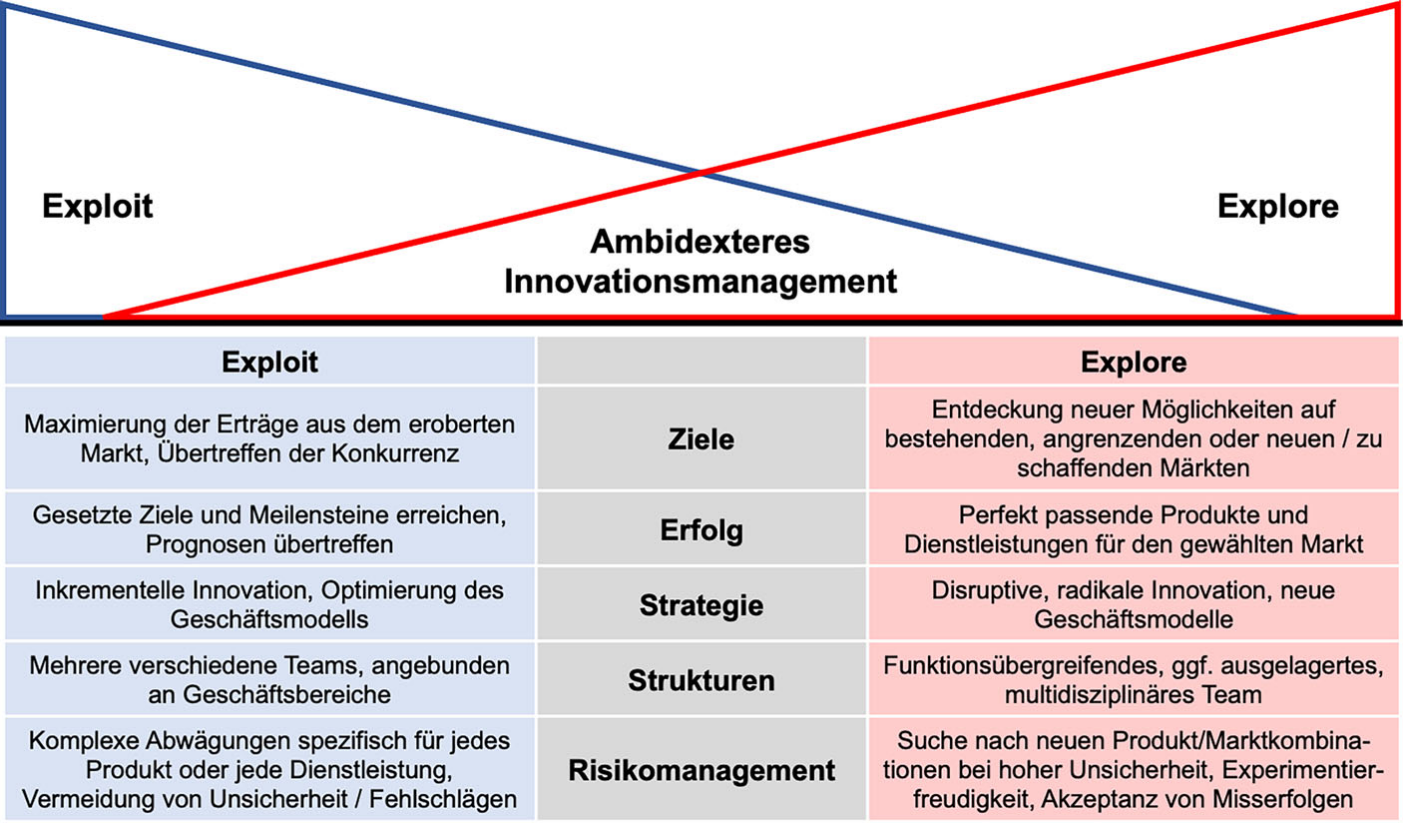
Ambidexteres Innovationsmanagement – Exploit vs. Explore

In letzter Zeit hat es sich, in Bezug auf die Innovationstätigkeit eingebürgert, die Kategorien des Ausnutzens bestehender Marktpositionen (*Exploit*) sowie der Auslotung neuer Gelegenheiten (*Explore*), sei es in Markt oder Technologie, zu unterscheiden. Abb. 1 zeigt die wesentlichen und im Weiteren nicht mehr dediziert aufgegriffenen Merkmale der beiden Modi – *Exploit* und *Explore* – auf (in Anlehnung an Humble et al. (2020), Osterwalder et al. (2020)).

Ambidextrie im Innovationsmanagement bedeutet dann, in diesen Aspekten beidhändig zu sein und *Exploit- *sowie* Explore-Modus *in ein Gleichgewicht zu bringen. Damit gilt es sowohl bestehende Märkte auszuschöpfen und sie mit weiter entwickelten Technologien und neuen Produkt- und Dienstleistungsmerkmalen für veränderte Bedürfnisse zu bedienen, aber gleichzeitig auch Risiken einzugehen und nach völlig neuen Geschäftsmodellen zu suchen, die beispielsweise Markt verändernde disruptive Technologien berücksichtigen. Bei bestehenden Ressourcen wird, wie in der Abbildung dargestellt, ein größerer Einsatz im Explorieren neuer organisatorischer Chancen dazu führen, dass weniger Kapazitäten für das Ausschöpfen des traditionellen Geschäfts verbleiben.

Das Gleichgewicht zwischen Exploit und Explore herzustellen, klingt einfach und stellt sich doch schwierig dar. Typische Ansätze in der Ambidextrie im Innovationsmanagement unterscheiden beispielsweise die **strukturelle Ambidextrie** von der Zeitlichen (z. B. Thinktank-Ambidextrie 2022). Die strukturelle Variante schafft gezielt parallele Strukturen neben der Unternehmensorganisation, um dann etwa in Spin-offs des Unternehmens die disruptive Innovation zu fördern. Die **zeitliche Ambidextrie** unterscheidet zwischen verschiedenen Phasen eines Unternehmens, in welchen entweder inkrementelle oder sprunghafte Innovationen sinnvoll sind. Beide Varianten sind gangbar, jedoch vermeiden sie, Exploitation und Exploration wirklich gemeinsam zu denken. Damit tauchen zum Beispiel Probleme auf, wenn der genaue Zeitpunkt des Übergangs von einem in einen anderen Innovationsmodus nicht einfach zu bestimmen ist, wenn ein Start-up in einen eroberten, regelmäßigen Markt übergeht, wenn über die Budgetverteilung zwischen den inkrementellen und disruptiven Innovationstätigkeiten diskutiert wird oder wenn die disruptive Innovation aus der abgegrenzten Teilorganisation in die Stammorganisation übertragen werden muss.

Als Alternative fordert die **kontextorientierte Ambidextrie** von Führungskräften, die richtigen Zeitpunkte, Organisationseinheiten oder Produkt- und Dienstleistungsgruppen für verschiedene Innovationsmodi zu identifizieren und diese dann entsprechend zu fördern. Führungskräfte sind damit aufgerufen zu lernen, zwischen den unterschiedlichen Innovationsmodi zu unter- und entscheiden sowie auch dazwischen wechseln zu können – und, was sicher nicht einfach sein wird, ihre jeweiligen Mitarbeitende auf diesem Weg mitzunehmen. Zunehmend mehren sich überdies die Anzeichen, dass es schwierig sein wird, den Kontext von Unternehmen oder ihren Einheiten eindeutig einem der Innovationsmodi zuzuordnen. So zeigte bereits das Horizon-Modell von McKinsey (2009) auf, dass einem zweiten Horizont der Innovationstätigkeit genau in der Mitte zwischen eher traditioneller Innovationstätigkeit (relativ kurzfristiger, erster Horizont) und disruptiver Innovationsarbeit (dritter, langfristiger Horizont) deutlich zu wenig Aufmerksamkeit der Unternehmensleitung zuteilwird. In diesem zweiten Horizont, nach etwa zwei bis fünf Jahren, sollten, nach Innovationen im Kerngeschäft, die insbesondere die Effizienz in Prozessen steigern (Horizont 1), beispielsweise Service-Innovationen entstehen, die das Kerngeschäft und das bestehende Geschäftsmodell anpassen. Die im folgenden Beitrag dargestellte Studie verstärkt die Befürchtung, dass es kaum einen Zeitpunkt oder eine Situation des Unternehmens oder von Unternehmenseinheiten gibt, in welchen nicht gleichzeitig Exploration und Exploitation notwendig sind.

Angesichts der Schwierigkeiten der zeitlichen, strukturellen und kontextuellen Ambidextrie unternehmen die Autorinnen in Kap. 4 den Versuch, das strategische „entweder-oder“ der beiden Innovationsmodi in den neben der Zielsetzung und Strategie wesentlichen Bereichen Unternehmenskultur, Führung und Digitalisierung in ein „sowohl-als-auch“ zu überführen.

Um verschiedene Innovationsmodi zu bedienen, muss sich das gesamte Unternehmen auf einen **Kulturwandel **einlassen. Im Vordergrund steht hier weniger der große Umbruch der bestehenden Kultur, ausgerichtet auf inkrementelle oder disruptive Innovation, in die jeweils andere. Wichtig vielmehr ist die Erkenntnis, dass man, auf Basis einer Kenntnis der kulturellen Unterschiede, das Neue tun kann, ohne das Alte lassen zu müssen. Es sind dann eher gezielte kleine Schritte, die es ermöglichen, den jeweils anderen Innovationsmodus parallel zuzulassen (Steiner und Landes 2022). **Führungskräfte** sind im sowohl-als-auch der Innovationsmodi aufgerufen, gezielt im Werkzeugkasten der Führung nach kleinen Veränderungen hin zu den jeweils für den anderen Innovationsmodus geeigneten Methoden zu suchen, ohne die traditionellen Ansätze über Bord zu werfen. Eine neue Kultur ebenso wie das neu geführte Miteinander im Unternehmen erfordert eine neue digitale Unterstützung. Geeignete Software, beispielsweise für unterschiedliche Formen der innerbetrieblichen Zusammenarbeit, kann Ambidextrie in der Innovationstätigkeit fördern. Dabei kann neue oder angepasste Soft- und Hardware im Arbeitsumfeld das Handeln, ob auf Führungs- und Entscheidungs- oder Mitarbeiterebene, leiten. Persuasiv – indem sie den Rahmen für digitale Aktivitäten vorgibt und damit das Verhalten prägt (Fogg 2002).

Beherrscht ein Unternehmen kulturell, in der Führung bis auf Mitarbeiterebene grundsätzlich beide Innovationsmodi und versteht ihre Chancen und Risiken, so können auch menschliche Barrieren oder Fehleinschätzungen überwunden werden. Führungskräfte beispielsweise fühlen sich (auch) in der kontextuellen Ambidextrie gefordert, in allen Situationen direkt klar zu sehen, wie sie zu bewerten sind und ihnen zu begegnen ist. Dies führt in den heutigen unsicheren, volatilen und komplexen Märkten potenziell zu übereilten oder falschen Reaktionen – sei es, weil man zu lange auf bewährten Geschäftsmodellen beharrt oder einen Markt zu rasch verlässt. Beharrt man zu lange, so ist häufig, wie etwa im Segment der Zeitschriften, die den Zuwachs an Informationsangeboten im Internet skeptisch betrachteten, die Sorge vor Selbstdisruption entscheidend. Auch kann der zu spät geplante Wechsel in neue Märkte davon geprägt sein, dass, wie im Bereich der Fotografie, das Potenzial neuer Technologien überschätzt wurde. Im Zusammenspiel von Exploit und Explore finden sich hier potenziell Lösungen, die ein neues Geschäftsmodell mit dem alten verbinden. Moderne Märkte verlangen gut balancierte Lösungen zur richtigen Zeit. Diese erfordern neben einer Ambidextrie im Innovationsmanagement auch **Ambiquitätstoleranz. **Sie bezeichnet die Fähigkeit eines Menschen – und damit auch einer Führungskraft – auch Situationen zu beherrschen, die unklar, unsicher und vielleicht sogar widersprüchlich sind. Sie fällt im Innovationsmanagement leichter, wenn man über das Werkzeug für die Exploitation und die Exploration aber auch für verschiedene Ausprägungen und auch Zwischenzustände verfügt.

Wie es gehen kann, insbesondere den Schnittbereich der beiden Modi Exploitation und Exploration zu bedienen, wie ihn Abb. 1 dargestellt, zeigen wir in Kap. 4 auf. Zuvor werfen wir noch einen Blick auf verschiedene Branchen. Denn obwohl nicht jede Branche gleichermaßen von aktueller und zukünftiger Disruption betroffen ist, werden dazu gehörige Unternehmen zwar beide Innovationsmodi beherrschen müssen, aber den einen oder anderen Innovationsmodus betonen.

## Anforderungen einzelner Branchen

**Abb. 2:**
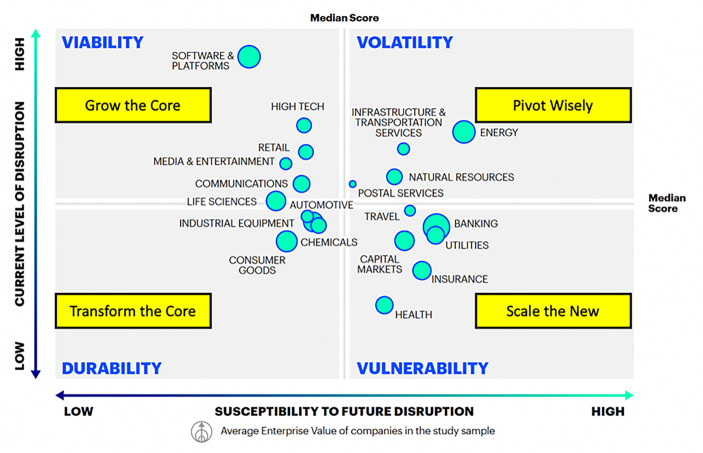
Disruptability Index Industry Sector Matrix (Accenture 2018)

Und plötzlich war es da: Das Start-up, welches eine ganze Branche verändert und etablierte Player obsolet gemacht hat! Ein Mythos, der sich wacker hält. Die Anfälligkeit einer Branche für disruptive Veränderungen wird jedoch nicht durch eher unvorhersehbare Ereignisse bestimmt, sondern kann recht präzise eingeschätzt werden. Dies hat Accenture auf einer Datenbasis von 3629 Unternehmen getan und den sogenannten *Disruptability Index* geprägt (Accenture 2018). Dieser umfasst zwei Dimensionen: Das *aktuelle* Disruptionsniveau einer Branche sowie ihre Anfälligkeit für Disruptionen *in der Zukunft.* Zur Messung des aktuellen Disruptionsniveaus wird Präsenz und Durchdringung von neuen, disruptiven Unternehmen sowie die finanzielle Leistungsfähigkeit der etablierten Unternehmen in einer Branche herangezogen. Die Anfälligkeit für zukünftige Disruptionen basiert auf drei Variablen: Die *operative Effizienz*, die *Innovationsstärke* und die *Fähigkeit zur Verteidigung der eigenen Position* der etablierten Unternehmen. Aus diesen beiden Dimensionen (0–1 Skala; 1 = most susceptible / disrupted) ergibt sich die 2 × 2 Matrix in Abb. 2.

Die vier Quadranten der Matrix weisen auf die Branchendynamik heute und in Zukunft hin: **Durability **(*Dauerhaftigkeit*),** Viability **(*Rentabilität*),** Volatility **(*Volatilität*) und **Vulnerability **(*Anfälligkeit*). Diese Dynamik wiederum gibt Hinweise auf die Unternehmensstrategie und auf die Konfiguration der Ambidextrie im Innovationsmanagement. Mithilfe dieser Matrix können sich Unternehmen oder einzelne Geschäftseinheiten einordnen und passende Strategien ableiten. Grundsätzlich gilt, dass Unternehmen beide Innovationsmodi gleichzeitig beherrschen müssen aber je nach Veränderungsdynamik des Marktes Schwerpunkte setzen. Auf dieser Basis entstehen dann ausgewogene und an die Situation des Unternehmens angepasste Innovationsportfolios, welche eine strategische Allokation des Innovationsbudgets auf inkrementelle und disruptive Innovationsaktivitäten ermöglichen.

Im Segment **Durability** finden sich etablierte Unternehmen, die lange am Markt bestehen und sich durch starke Marken, weltweite Marktzugänge und integrierte Wertschöpfungsketten auszeichnen. Durch diese strukturellen Vorteile sind diese Unternehmen aktuell selten von neuen Marktteilnehmenden bedroht. In der Studie fallen 11 % der untersuchten Unternehmen, zum Beispiel Maschinenbauunternehmen und Automotive OEMs (*Original Equipment Manufacturer*, deut. Originalausrüstungshersteller), in dieses Segment. Im Innovationsmanagement sollten sich diese Unternehmen auf die proaktive Umgestaltung des Kerngeschäfts fokussieren, also den Exploit-Modus priorisieren. Ziel sind wettbewerbsfähige Kostenstrukturen und Effizienzvorteile im Kerngeschäft, um die Rentabilität weiter zu steigern. Dadurch jedoch sind diese Unternehmen dann auch in der Lage, mit daraus resultierenden Investitionskapazitäten massiv mit neuen, teils disruptiven Geschäftsfeldern zu experimentieren. Accenture nennt diese Innovationsstrategie „**Transform the Core**“.

Unternehmen, die sich im Segment **Viability** befinden, mussten sich in den letzten Jahren gegen starken Wettbewerb neuer Player am Markt behaupten und auch jetzt verzeichnen die Branchen einen hohen Grad an radikaler Veränderung. Vertreter dieses Segments sind Software- und Medienunternehmen oder der Handel und machen ca. 18 % der von Accenture untersuchten Unternehmen aus. Der starke Wettbewerb in Kombination mit vielen neuen Disruptoren hat hohe Innovationsraten zur Folge. Die dadurch erreichten Wettbewerbsvorteile sind jedoch aufgrund der Marktdynamik meist nur von kurzer Dauer. Die Innovationsstrategie „**Grow the Core**“ bedeutet, dass Unternehmen in dieser Situation Maßnahmen zum Aufbau neuer Fähigkeiten, wie digitales Marketing oder Künstliche Intelligenz, ergreifen müssen, um ein konstantes Innovationsniveau zu halten. Auf dieser Basis entstehen neue innovative Angebote, die bei bestehenden Kundinnen und Kunden auf Interesse stoßen und so zusätzliche Wachstumsquellen für das Kerngeschäft ermöglichen. Im Explore-Modus liegt der Fokus auf einer aggressiven Expansion in angrenzende oder völlig unbekannte Märkte. Dies wird durch die Stärke des Kerngeschäfts ermöglicht.

33 % der untersuchten Unternehmen, etwa aus den Bereichen Infrastruktur, Energie oder Telekommunikation, wurden in besagter Studie dem Segment **Volatility** zugeordnet. Hier bestimmt eine hohe Veränderungsdynamik heute und eine noch höhere in Zukunft die Überlebensfähigkeit. Ehemals starke Eintrittsbarrieren sind durch Marktliberalisierungen und neue technologische Möglichkeiten erodiert, was von neuen Marktteilnehmenden ausgenutzt wird und dazu beiträgt, dass sowohl Umsätze als auch Gewinnmargen der etablierten Unternehmen in diesem Segment sinken. Neue Mobilitätsdienstleister wie Uber benötigen im Vergleich zu etablierten Anbietenden keine eigene Fahrzeugflotte, um vergleichbare oder gar höherwertige Dienstleistungen anbieten zu können. Anstatt das Kerngeschäft einfach aufzugeben, müssen etablierte Unternehmen in diesem Marktumfeld ein gut austariertes Gleichgewicht finden. Die „**Pivot Wisely**“-Strategie sieht eine Balance zwischen dem Exploit- und Explore-Modus vor, denn wenn Unternehmen zu schnell vom Kerngeschäft zu den neuen Geschäftsfeldern wechseln, laufen sie Gefahr, zu schnell finanziell auszutrocknen. Wenn sie sich zu langsam umorientieren, besteht die Gefahr, dass sie insgesamt überflüssig werden. Zentral ist im Sinne der Ambidextrie zu erkennen, in welche aktuellen Geschäftsbereiche und Geschäftsmodelle man weiterhin investiert und auf welche eher disruptiven Innovationschancen man zukünftig bauen will. Ein ausbalanciertes Portfolio der Innovationsaktivitäten ist für Unternehmen in dieser Situation entscheidend.

Die **Vulnerability**-Situation kann kurz mit „die Ruhe vor dem Sturm“ beschrieben werden. Betroffen sind beispielsweise Versicherungen, Banken oder das Gesundheitswesen. Dabei handelt es sich um etablierte Unternehmen, die heute noch von hohen Markteintrittsbarrieren, zum Beispiel strenge regulatorische Vorgaben oder hohe Kapitalanforderungen, profitieren. Um die starke Marktposition zu sichern, sollten Unternehmen in diesem Segment ihr Kerngeschäft optimieren, indem sie neue Technologien und Data Analytics nutzen, um ihren Kundinnen und Kunden verbesserte Produkte und Dienstleistungen anzubieten. Wenn die Kompetenz in den eigenen Reihen fehlt, sollte diese gezielt akquiriert werden. Banken kaufen etwa gezielt Fintech Start-ups, um Zugang zu neuen Technologien und notwendigen Kompetenzen zu erhalten. Gleichzeitig sollten Unternehmen an skalierbaren neuen Ideen – „**Scale the New**“ – arbeiten. Am besten, im Sinne einer strukturellen Ambidextrie, mithilfe einer spezialisierten Einheit, wie ein Innovationslabor oder eine Digitale Fabrik, um wertvolle Innovationen unabhängig vom Tagesgeschäft realisieren zu können.

Man sieht deutlich, dass die Beidhändigkeit im Innovationsmanagement in allen dargestellten Phasen der Disruption relevant ist. Egal welche Strategie ein Unternehmen wählt – ob „Transform the Core“ oder „Scale the New“ – es gilt immer, die richtige Balance zwischen Exploitation und Exploration zu finden und Innovationsbudgets so zu verteilen, dass je nach Marktdynamik beide Innovationsmodi – inkrementell und disruptiv – ausreichend gefördert werden.

## Ambidextrie im Innovationsmanagement verankern und leben

### Ambidextere Kultur

Ambidextrie spiegelt sich in der Unternehmenskultur in den zwei Modi Exploit und Explore wider, wobei beide Modi grundsätzlich existieren, aber der eine oder andere Modus ausgeprägter sein kann. Im Exploit-Modus arbeiten Mitarbeitende nach klaren Regeln und Prozessen, was beispielsweise eher in der Produktion und in der Logistik gefordert wird. Im Explore-Modus haben Mitarbeitende dagegen Freiheiten, um in ihren Arbeitsprozessen kreativ zu sein, wie es etwa in Design-Studios oder Werbungsagenturen der Fall ist. In den einzelnen Branchen sind Muster fest verankert, die eher die eine oder die andere Ausprägung der zwei Modi betonen. Dennoch gibt es zum Beispiel Instrumente, die dazu anleiten, Innovationsmöglichkeiten in bestehenden Prozessen, also im Exploit-Modus, auszuloten (z. B. der Einsatz von Blockchain und Künstliche Intelligenz in der Logistik (Goudz et al. 2021)).

Beide Modi zu beherrschen und sie gezielt komplementär und in einer Parallelität einzusetzen, ist jedoch herausfordernd und selten einfach umsetzbar. Stattdessen wird ein Bewusstsein darin gefordert, wann im Unternehmen oder spezifisch in einem Fachbereich ein Modus ausgeprägter gelebt werden soll, ohne den anderen Modus ganz wegzulassen, und wann beide Modi etwa gleich eingesetzt werden sollen. Wird der Fokus etwa auf die Prozessoptimierung im Onboarding eines Neueintritts gelegt (Exploit), so können dennoch Aktivitäten in der Evaluation neuer technischer Lösungen (z. B. KI-basierte Workflow-Tools) geprüft werden (Explore), um die Prozessoptimierung mit neuen Technologien zu unterstützen. Gleichwohl könnte aber auch entschieden werden, dass neben der Prozessoptimierung der Schwerpunkt auf den Einsatz neuer Technologien gelegt wird, wodurch die explorativen Aktivitäten zunehmen.

**Abb. 3:**
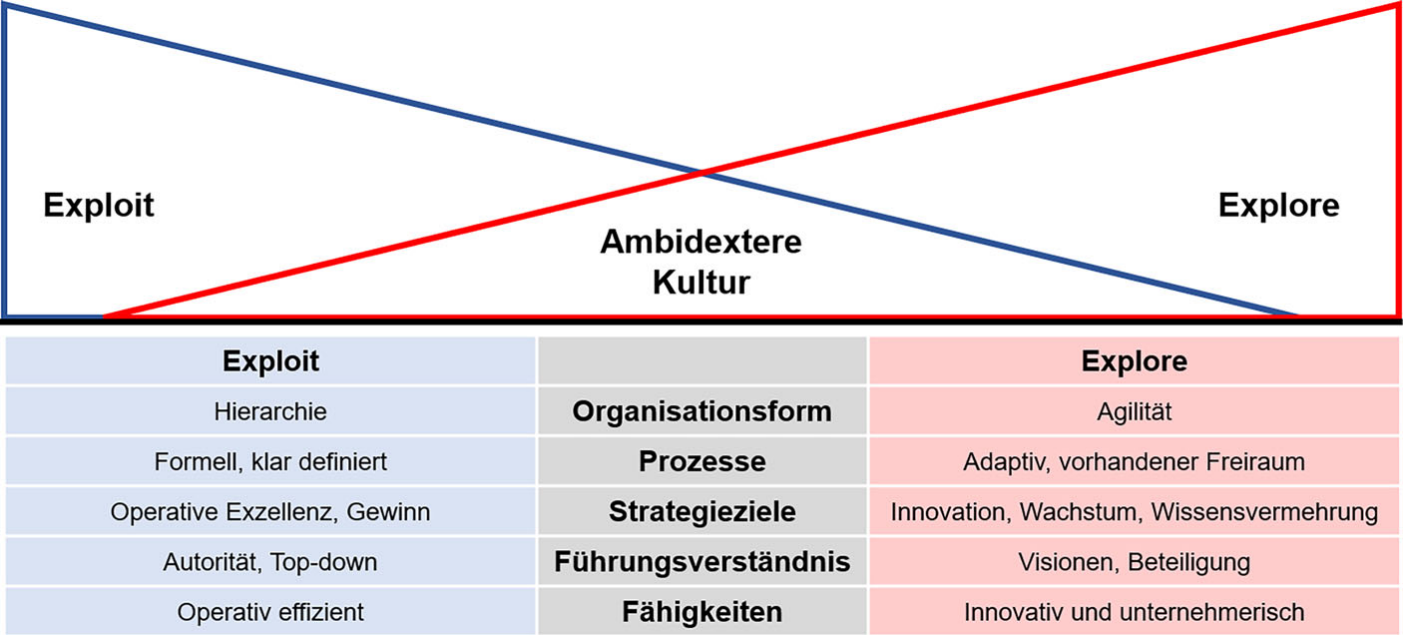
Ambidextere Kultur – Exploit vs. Explore

Eine Unternehmenskultur zeichnet sich durch verschiedene Ausprägungen aus, die anhand der beiden Modi in Abb. 3 (in Anlehnung an O’Reilly und Tushman 2004) visualisiert und in den nachfolgenden Abschnitten näher beleuchtet werden. Verschiedene Faktoren der zwei Modi – Exploit und Explore – beeinflussen somit die Unternehmenskultur, die wiederum einen Einfluss auf das Innovationsmanagement hat.

Die **Organisationsform** und ihre **Prozesse **haben einen Einfluss darauf, wie Mitarbeitende sich in einem Unternehmen bewegen dürfen. Hierarchische Unternehmen, wie etwa Behörden oder das Militär, zeichnen sich durch klar definierte Prozesse und Strukturen aus. Diese strikten Vorgaben legen wiederum fest, wie die jeweiligen Arbeitsprozesse und Mitarbeitende innerhalb des Unternehmens organisiert sind. Entscheidungsbefugnisse sind für bestimmte Führungskräfte vorbehalten. Damit herrscht für Mitarbeitende wenig Raum für eigenverantwortliches Handeln. Somit haben hierarchische Unternehmen oft Schwierigkeiten, ein Innovationsmanagement aufzubauen und innovative Ideen wegen langen und starren Prozessen der Bürokratie zeitnah und erfolgreich umzusetzen. Führungskräfte müssen deshalb die Bedeutung von Innovationen anerkennen und eine Kultur einführen, die den nötigen Freiraum für Innovationen schafft (mehr dazu im Abschn. 4.2).

Agile Unternehmen, deren Geschäftsmodell bereits auf Innovation beruhen (z. B. Tech-Start-ups wie Uber, Zoom oder Spotify), haben einfachere Bedingungen für den Aufbau und das Führen eines Innovationsmanagements, da ihre Organisationsform bereits darauf ausgelegt ist, schnell und flexibel auf Veränderungen zu reagieren. Mit flachen Hierarchien sind Entscheidungsprozesse schneller, und es können Mechanismen eingesetzt werden, um Mitarbeitende, etwa für die Sammlung von Ideen für konkrete Innovationsprojekte, mit einzubeziehen. Spotify hat beispielsweise ein internes Innovationsprogramm namens „Hack Week“, bei welchem Mitarbeitende eine Woche lang an Innovationsprojekten arbeiten können. Dabei ist es egal, ob die Projekte einen direkten Bezug zur alltäglichen Arbeit haben oder nicht. Mit dieser Maßnahme können einerseits neue Ideen und Innovationen gefördert und andererseits das Engagement der Mitarbeitende gestärkt werden (Spotify 2023). Die flexiblen und adaptiven Strukturen und Prozesse schaffen für Mitarbeitende einen gewissen Freiraum, um ihre Kreativität zu entfalten. Das bedeutet aber nicht, dass die Strukturen und Prozesse völlig lose sind. Im Gegenteil: auch in agilen Umgebungen gibt es klare Leitplanken und Rahmenbedingungen oder müssen noch geschaffen werden, innerhalb derer sich Mitarbeitende bewegen dürfen. Beispiele sind gemeinsame Prinzipien, wie Integrität oder klar definierte Ziele oder strategische Ausrichtungen des Unternehmens. Dies gewährleistet einerseits eine gewisse Stabilität, im Sinne von, dass die Mitarbeitenden wissen, welche Ziele das Unternehmen verfolgt und welche Handlungen von ihnen erwartet werden. Andererseits wird Raum für Kreativität geschaffen, da innerhalb dieser Leitplanken die Mitarbeitenden eigenverantwortlich handeln können, um innovative Lösungen zu entwickeln. Des Weiteren setzen agile Unternehmen eher auf cross-funktionale Teams, welche verschiedene Ideen, Perspektiven und Fähigkeiten in das Unternehmen mitbringen.

Naheliegend haben die **Strategieziele** einen Einfluss auf den Bewegungsraum der Mitarbeitenden. Eine Strategie nach operativer Exzellenz und Gewinn ist auf die Verbesserung bestehender Produkte, Dienstleistungen und Prozesse ausgerichtet. Kosteneinsparungen und Effizienzsteigerungen stehen im Fokus, um den Gewinn des Unternehmens zu maximieren. Innovationen konzentrieren sich in erster Linie auf inkrementelle Verbesserungen, um bestehende Geschäftsmodelle zu optimieren (z. B. Digitalisierung analoger Prozesse, wie digital formularbasierte Anträge). Mitarbeitende sind dazu angehalten, digitale Lösungen zu finden, um schnelle Ergebnisse zu erzielen. Bei einer Strategie nach Wachstum und Wissensvermehrung setzen Unternehmen auf disruptive Innovationen, um neben den Produkten, Dienstleistungen und Prozessen das Unternehmen weiterzuentwickeln. In diesem Kontext werden häufig Innovationsökosysteme als die Plattform für den Wissensaustausch genannt. Ein Innovationsökosystem ist ein Netzwerk von verschiedenen Akteuren (Unternehmen, Start-ups, Hochschulen, etc.), die zusammenarbeiten, um Innovationen zu schaffen, zu entwickeln und zu verbreiten. Es fördert den Austausch von Ideen und Ressourcen sowie die Zusammenarbeit zwischen den Akteuren, um Innovationen schneller und effektiver zu entwickeln, zu testen und zu skalieren (Junker und Büdding 2022). Silicon Valley ist dabei eines der bekanntesten Innovationsökosysteme. Eine solche Strategie erfordert jedoch von allen Mitarbeitenden und Führungskräften ein offenes Denken für Veränderungen und deren Akzeptanz.

Ein **Führungsverständnis**, das auf Autorität und Top-down-Entscheidungsprozessen basiert, kann die Innovationsarbeit bremsen. Wenn bestimmte Führungskräfte die alleinige Entscheidungsbefugnis haben und Innovationen von oben herab angeordnet werden, kann dies zu einer Abwesenheit von kreativen Ideen und einer fehlenden Akzeptanz von Veränderungen führen. Die Entscheidungen und das Verhalten der Führungskräfte können somit einschränkende Einflüsse auf die Kreativität und das Engagement der Mitarbeitenden haben. Des Weiteren tolerieren Personen mit einem autoritären Führungsverständnis und einem strikten Kontrollwunsch kaum, dass ihre Mitarbeitenden Risiken eingehen oder sogar Fehler machen. Ein Führungsverständnis, das hingegen auf Visionen und Beteiligungen basiert, fördert Innovationstätigkeiten im Unternehmen. Kommunizieren die Führungskräfte klare Visionen, die auf gemeinsamen Werten und Prinzipien basieren (sog. Leitplanken, s. oben) und ermöglichen die Führungskräfte, dass Mitarbeitende die Ressourcen für Innovationsarbeiten erhalten, so unterstützt dies zum einen die Kreativität und das Engagement der Mitarbeitenden und zum andern können mithilfe von Innovationen Unternehmensziele wie zum Beispiel „die Marktkraft ausbauen“ erzielt werden.

Mitarbeitende, die in einer exploit-orientierten Umgebung arbeiten dürfen, zeichnen sich durch **Fähigkeiten** mit operativer Effizienz aus. Sie haben ein gutes Verständnis für die Unternehmensprozesse und wissen, diese zu analysieren, optimieren und ggf. mit digitalen Lösungen zu automatisieren. Dabei stehen unterschiedliche Ziele im Fokus, wie die Produktivität im Unternehmen zu steigern, eine hohe Qualität der Produkte und Dienstleistungen zu gewährleisten und die eigenen Ressourcen und die des Unternehmens effizient zu nutzen. Mitarbeitende, die in einer explore-orientierten Umgebung arbeiten dürfen, haben hingegen unternehmerische (sog. „entrepreneurial“) Fähigkeiten. Die kreative, innovative Lösungsfindung für unterschiedlich komplexe Probleme, die Fähigkeit sich adaptiv auf neue Situationen einzustellen sowie die Eigenverantwortung und die intrinsische Motivation das Unternehmen voranzubringen prägen die heutigen „Entrepreneurs“. Beide Fähigkeitsklassen sind für den Erfolg eines Unternehmen wichtig. Mitarbeitende sind in einer ambidexten Kultur in der Lage, sowohl operative als auch unternehmerische Fähigkeiten zu entwickeln und zu nutzen. Eine operative Effizienz trägt dazu bei, dass ein Unternehmen effizienter arbeitet, während unternehmerische Fähigkeiten darauf einzahlen, dass ein Unternehmen am Puls der Zeit bleibt.

Wenn innerhalb eines Unternehmen die Akzeptanz vorhanden ist, dass Mitarbeitende in einer exploit-orientierten Umgebung „andere Spielregeln“ als Mitarbeitende in einer explore-orientierten Umgebung haben (und umgekehrt), so wird das Unternehmen in der Lage sein, eine Kultur zu leben, die es ermöglicht, weiterhin im Markt zu bestehen (Thinktank-Ambidextrie 2022). Somit ist das Ziel jedes Unternehmens, beide Innovationsmodi zu vereinen und dass diese von den Mitarbeitenden und den Führungskräften gelebt werden.

### Ambidextere Führung

In der Führung zeigt sich Ambidextrie zunächst im Sinne ihrer kontextorientierten Form darin, dass die Führungskraft zwischen zwei Führungskonzepten wechseln kann, die entweder den Exploit- oder den Explore-Modus unterstützen (z. B. Thinktank-Ambidextrie 2022). Ambidextrie kann jedoch auch darin bestehen, diese Führungsmodi in einzelnen Aspekten zu verflechten oder zusammenzubringen. Denn Führung von Mitarbeitenden gelingt nur, wenn gemeinsame Prinzipien, zum Beispiel in Bezug auf Offenheit, Delegation, Mitwirkung oder Verantwortung geteilt werden. Ein Wechsel der Führung von einem bekannten in einen anderen Modus oder, noch schwieriger, zwischen verschiedenen Modi, ist fast unmöglich, wenn nicht die grundsätzlichen Prinzipien beider Modi zumindest bekannt, verstanden, angelegt und in gewisser Weise von allen Beteiligten gelernt sind. Wo sind also Stellschrauben, die verschiedene Formen der Führung aneinander annähern, um die Organisation mit ihren Mitarbeitenden kulturell zu befähigen, im Innovationsmanagement verschiedene Modi je nach Situation und Bereich einzunehmen?

**Abb. 4:**
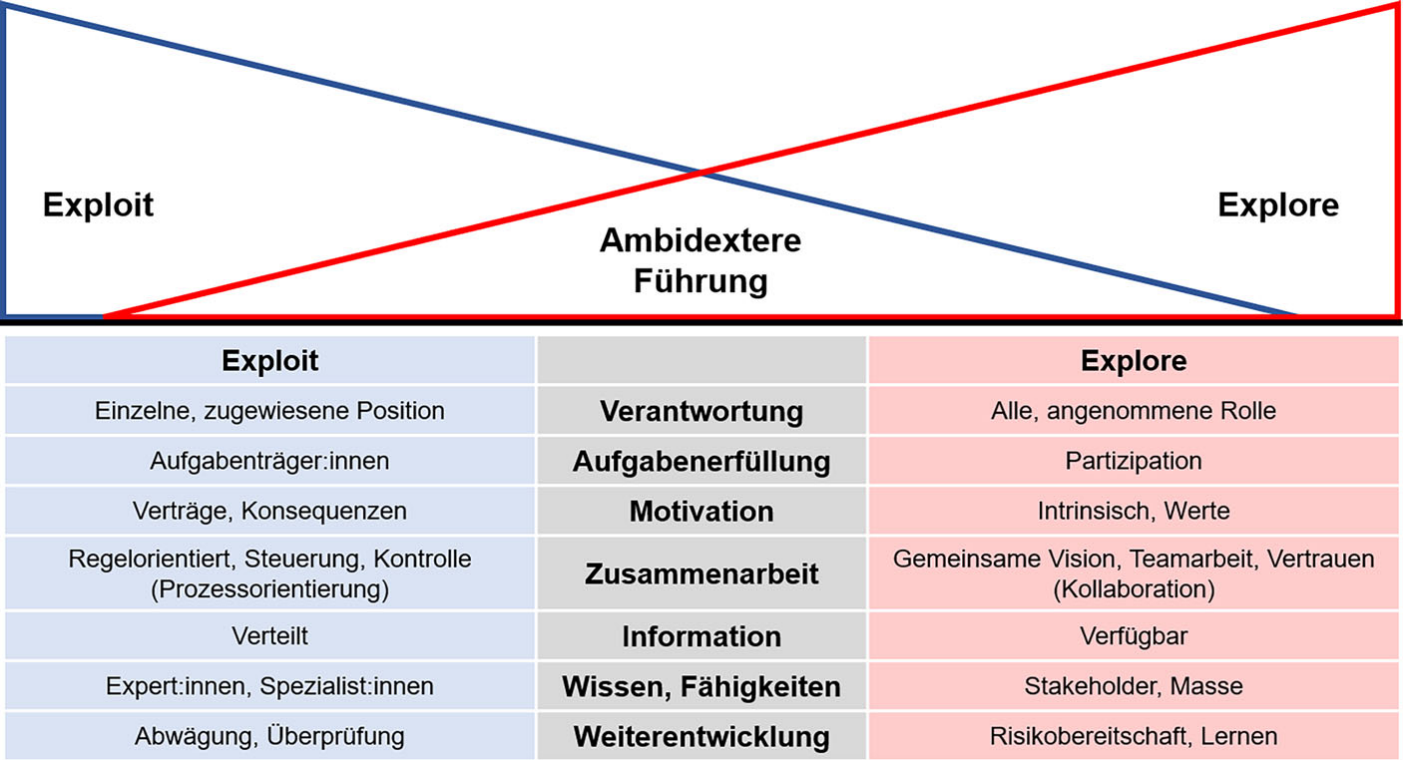
Ambidextere Führung – Exploit vs. Explore

Verschiedene Führungsformen zeichnen sich durch unterschiedliche Führungsverständnisse, Führungsstile oder Managementtechniken aus und hängen zudem von Organisationsformen ab. Im Folgenden soll dies nicht lehrbuchhaft hergeleitet und auch nicht gezielt auf aktuelle Entwicklungen eingegangen werden. Stattdessen werden im Folgenden, wie in Abb. 4 dargestellt, wesentliche und zwischen Exploitation und Exploration differenzierende Merkmale der Führung (nach Van Dick et al. 2016 und Thinktank-Ambidextrie 2022) herausgegriffen und hinsichtlich ihrer Möglichkeiten geprüft, die beiden Innovationsmodi zu verbinden.

Der Unterschied zwischen einem Exploit- und einem Explore-Modus im Innovationsmanagement beginnt damit, ob und wie eine Führungskraft definiert wird. In der Tradition des Exploit-Modus wird die *Position* des Innovationsmanagements in Unternehmensorganisationen zugewiesen, die damit die **Verantwortung** über die Innovationstätigkeit innehat. Ein Explore-Modus, wie beispielsweise in Start-ups, ist dadurch gekennzeichnet, dass sich *alle* oder zumindest viele Mitarbeitende, vielleicht in unterschiedlichen Bereichen für stetige und auch radikale Innovation verantwortlich fühlen. Auf dem Weg des Start-ups zu einer größeren Organisation wird das Start-up sich über die Zeit Gedanken darüber machen müssen, eine gewisse organisatorische Struktur anzunehmen und das Innovationsmanagement nicht mehr allen Mitgliedern zu überlassen, sondern die Führung in diesem Bereich zumindest temporär personell zu begrenzen. Dabei wird jedoch auf das Prinzip verzichtet, eine Position zuzuweisen, sondern vielmehr übernehmen Mitarbeitende in gemeinsamer Abstimmung die entsprechende *Rolle.*

Versucht das eher traditionelle Unternehmen im Sinne einer stärkeren Exploration die Verantwortung im Innovationsmanagement auf mehr Köpfe als die entsprechende Position zu verteilen, dann kann es damit beginnen, über die Verteilung der **Aufgabenerfüllung** in einer Organisation nachzudenken. Typischerweise sieht diese so aus, dass feste Aufgabenträgerinnen und Aufgabenträger, zum Beispiel im Innovationsmanagement (mit seiner Leitung und den entsprechenden Mitarbeitenden), die ihnen zugewiesenen Aufgaben, eben die Innovationstätigkeit, in Gänze übernehmen. Bereits vor einiger Zeit stellte sich heraus, dass einzelne Aufgaben in der Innovation auch Kundinnen und Kunden übertragen werden können (*Open Innovation*, z. B. Reichwald und Piller 2009). So lässt beispielsweise McDonalds seine nächsten Burger von Kundinnen und Kunden entwickeln oder Henkel überträgt ihnen die Erfindung einer Alternative zum Pritt-Stift. Wird dieses Konzept nicht allein als Marketinginstrument gesehen, so öffnet es den Weg zu einer umfassenden *Partizipation*, der breiten Beteiligung von Stakeholdern. Das Unternehmen sollte hierbei vor allem das Potenzial der Mitarbeitenden nicht übersehen und sie in Innovationsaktivitäten, für interne und externe Produkt‑, Dienstleistung‑, Prozess- und Service-Innovationen, einbeziehen. Dabei beginnt und endet diese Integration nicht mit der Ideenfindung, sondern bezieht auch ein, vorab Risiken für das Geschäft des Unternehmens zu erkennen, die gefundenen Ideen zu bewerten, Entscheidungen zu treffen und Innovationen verantwortungsvoll weiterzutreiben. Hierzu bietet zum Beispiel Hyve eine Digital Innovation Toolbox, die neben einer offenen Ideenplattform (Hyve Crowd) weitere Tools zum Management von Ideen, zu ihrer Bewertung, zum Prototyping bis hin zum Test umfasst. Diese digitalen Tools ermöglichen mehr Exploration durch den Einbezug von mehr Mitarbeitenden. Jedoch auch einem Unternehmen, das zu einem mehr an Exploitation, und damit zu einer besseren Ausschöpfung von neuen Ideen gelangen möchte, können digitale Tools im Innovationsmanagement helfen, indem sie die Grundsätze, verschiedenen Aktivitäten und Prozesse einer gemeinsamen Innovationstätigkeit sammeln und in einer digitalen Form zusammenführen.

Wesentlich für das Gelingen der Partizipation von Mitarbeitenden ist jedoch, dass weitere Grundlagen des Zusammenarbeitens geschaffen oder verändert werden. So basiert beispielsweise die traditionelle Arbeit im Exploit-Modus auf Verträgen oder vertragsähnlichen Vereinbarungen über Tätigkeiten und zu erreichende Ziele, die mit Mitarbeitenden geschlossen werden und die, zur **Motivation** zielgerichteten Arbeitens, jeweils mit Konsequenzen hinsichtlich der Vertragserfüllung versehen sind. Es ist eine einfache Weisheit, dass einzelne Zielvorgaben den Blick auf ggf. im Vorfeld nicht als so wichtig erscheinende Ziele verstellen und/oder Tätigkeiten verhindern, die der Unternehmensvision dienlich gewesen wären. Für ein exploratives, auf neue Wege ausgerichtetes Handeln im Innovationsmanagement ist es daher wichtig, Mitarbeitenden dazu zu verhelfen, aus *intrinsischer* Motivation zu agieren. Nur Mitarbeitende, die sich mit dem, was ihr Unternehmen tut und ihrem eigenen Anteil daran identifizieren, werden gute Ideen für Innovationen entwickeln. Unternehmensvisionen und -werte sollten daher transparent, aber auch mit eigenen Werten von Mitarbeitenden abgeglichen sein.

Die Art der **Zusammenarbeit** schließt sich an die gemeinsame Zielstellung an. Im Exploit-Modus mit der eher extrinsischen Motivation entstehen aus den Zielvorgaben (hierarchische) *Regelwerke*, wie Prozessvorgaben, mit welchen Mitarbeitende in ihrer Arbeit gesteuert und ihre Tätigkeit sowie Ergebnisse kontrolliert werden. Der als *Kollaboration* bezeichnete Modus der Zusammenarbeit, so wie er sich eher im Explore-Modus zeigt, stellt die an *gemeinsamen Visionen* ausgerichtete, *gleichberechtigte* und *verantwortungsvolle* Arbeit in selbst gewählten Teams unter der Prämisse des *Vertrauens* in den Vordergrund. Für den Weg hin zu mehr Kollaboration ist es, abgesehen von der Etablierung von Arbeit in einzelnen, interdisziplinären Teams oder Projekthäusern, ebenso wichtig, die *Kollaborationskompetenz* bei Führungskräften ebenso wie bei Mitarbeitenden zu schärfen, sodass ein Wechsel in diesen Modus überhaupt möglich ist. Vertrauen ist die Basis, jedoch ist diese nur schwer einzufordern. Für Führungskräfte bedeutet Kollaboration eine gemeinsame Vision zu fördern und vor allem Mitarbeitende als gleichberechtigte Partner anzuerkennen. So sollten sie beispielsweise im Kollaborationsmodus davon absehen, Aufgaben zuzuweisen oder den Ablauf der gemeinsamen Arbeit vorzugeben. Bereits ein vorgegebener Meilenstein kann das Team davon abhalten, die Verantwortung für ihre Aufgabe zu übernehmen. Mitarbeitende können und müssen eigene Verantwortung übernehmen und erkennen, dass es nicht auf ihre Arbeit als Einzelkämpfer, sondern auf das untereinander verbundene Arbeiten sowie das gemeinsame Ergebnis ankommt und sich freiwillig dafür einsetzen (Robra-Bissantz und Siemon 2019).

Es ist ebenso schwierig, vom Exploit-Modus der Prozessorientierung in mehr Kollaboration zu gelangen, wie umgekehrt. Eine Hilfestellung in beide Richtungen ist es nach neueren Erkenntnissen, beispielsweise der Holakratie in Unternehmen (www.holacracy.org), zwar die inhaltliche, an der Geschäftstätigkeit ausgerichtete Arbeit frei, dezentral und gleichberechtigt zu gestalten, aber für die Koordination derselben, und damit für das (gemeinsame) Einbringen von Vorschlägen, für die Arbeitsverteilung oder gemeinsame Entscheidungen ebenso wie für die Evaluation des Erreichten feste Strukturen vorzugeben: zum Beispiel in formalisierten oder methodisch gestützten Workshops und Meetings oder über eine digitale Kanban-orientierte Teamarbeit. Damit kann, über vorab festgelegte eher strukturierte oder freie Phasen und Aktivitäten, jeweils vom eher explorativen in den exploitativen Modus und zurück gewechselt werden.

Weitere Grundlagen, die zum einen den Wechsel von der Exploitation zur Exploration ermöglichen und zum anderen aber auch den Explore-Modus in Richtung der effizienten Ausnutzung von erreichten Positionen stärken, liegen in der verbesserten Nutzung von **Informationen, Wissen und Kompetenzen**. Die *Informationsversorgung* im traditionellen Unternehmen erfolgt über gezielte Verbreitung oder Weitergabe an diejenigen, die sie aufgrund ihrer Aufgabenbeschreibung oder Position im Unternehmen benötigen. Dazu treten inoffizielle Informationsflüsse, die Informationen zufällig oder aufgrund persönlicher Vernetzung verbreiten – aber damit nicht alle erreichen. In Partizipations-Szenarien ebenso wie in der an gemeinsamen Zielsetzungen ausgerichteten Arbeit benötigen alle Mitarbeitenden potenziell Informationen über alles, was das Unternehmen betrifft, sei es die Geschäftstätigkeit, externe Bedingungen, Märkte oder die eigenen Ressourcen und Kompetenzen. Damit sollte eine entsprechende Transparenz immer gewährleistet sein, wobei die Art der Informationsversorgung durchaus in einem eigenverantwortlichen Hol-Modus von verfügbaren statt verteilten Informationen abgebildet sein kann. Ein modernes, beispielsweise als Wiki aufgebautes digitales Wissensmanagementsystem kann dies leisten. Zudem unterstützt es aber auch das explorierende Start-up, wenn hier die Mitarbeitenden ihre neuen Erkenntnisse rasch und einfach beitragen können.

Zunehmend zeigt sich, dass für das Unternehmen relevante Wissen und wichtige Fähigkeiten von Mitarbeitenden nicht mehr allein von ihrer formalen Bildung oder ihrer derzeitigen Position im Unternehmen abhängen und sich daraus auch nicht mehr ableiten lassen. Um Chancen der Exploration zu bergen, ist es daher wichtig, nicht allein auf Expertinnen und Experten oder Spezialistinnen und Spezialisten zu vertrauen, die man entsprechend ihres Bildungsabschlusses oder aus ihrer bisherigen Karriere identifiziert. Stattdessen gilt es, breit über die Masse sowie über verschiedene Stakeholder auch eher weiche Kompetenzen wie Kreativität oder informelles Wissen zum Beispiel über neue Märkte gezielt zu nutzen und zu fördern.

Ambidextrie in der **unternehmerische Weiterentwicklung **bedeutet für das explorierende Unternehmen ein mehr an Überprüfung und Abwägung von Risiken. Damit gilt es, neue Ideen nicht nur zu entwickeln und auf Märkten anzubieten, sondern diese auch zu evaluieren – und bestenfalls parallel zur Ideenentwicklung die Überprüfung des Erfolgs mitzudenken. So untermauert beispielsweise Flixbus seine neuen Dienste und strategischen Entscheidungen regelmäßig und fortlaufend auf seiner Datenbasis zum vergangenen Geschäft. Für das exploitierende Unternehmen erfordert die heute komplizierte, unsichere und sich rasch wandelnde Unternehmensumwelt zunehmend passende unternehmerische Reaktionen, die eine Weiterentwicklung mit disruptiven Ideen ermöglichen: bewusst Risiken einzugehen und gemeinsam immer weiter zu lernen. Einem Unternehmen im Exploit-Modus ein wenig mehr Risikobereitschaft statt Risikoabwägung zu empfehlen, wird schwierig sein. Gemeinsames Lernen jedoch ist möglich – und kann zudem die Abschätzung von zuvor als zu hoch bewerteten Risiken verbessern. Dazu gehört jedoch, auf Führungs- ebenso wie auf Mitarbeiterebene, die Einsicht, dass die stetige Weiterentwicklung angesichts des sich ständig verändernden Wissens eine dringende Notwendigkeit ist und nicht davon zeugt, einen Kompetenzerwerb verpasst zu haben.

### Ambidextrie und Digitalisierung

Im Kontext der Ambidextrie stellt die Digitalisierung sicher, dass Unternehmen Innovationsaktivitäten sowohl im Exploit- als auch im Expore-Modus durchführen können – je nach aktuellen Handlungsbedarf. Betrachten wir die Entscheiderebene in der heutigen digitalen Transformation, können wir zwei grundlegende Typen unterscheiden: Der **Bedachte**, der beobachtet und zunächst abwartet, wie sich neue Technologien entwickeln und eine gewisse Reife erlangen, und der **Mutige**, der schnelle und riskante Entscheidungen hinsichtlich Exploration und Einführung neuer Technologien trifft. Untersuchungen zu Resilienz von Unternehmen zeigen, dass eine reine Verteidigungshaltung tendenziell zu einer mittleren Unternehmensleistung führt, während eine reine Angriffshaltung eine Mischung aus gelegentlichen Siegen und einigen katastrophalen Misserfolgen liefert (McKinsey 2020). Ambidextrie soll diese beiden Welten zusammenbringen und balanciert exploitative und explorative Innovationsaktivitäten im technischen Umfeld, je nach Dynamik im Markt heute und in Zukunft.

Für die verschiedenen Innovationsmodi ist die Datenbasis sowie die damit verbundene Unsicherheit unterschiedlich. Der Exploit-Modus zielt auf die Maximierung der Erträge aus dem eroberten Markt. Die Datenbasis bezieht sich auf das aktuelle Geschäft: Wettbewerb, Marktentwicklung, Umsatzzahlen. Im Explore-Modus müssen Unternehmen (unbekannte) Bedarfe in der Zukunft prognostizieren. Ob diese Zukunftsszenarien wirklich eintreten, ist unsicher. Umfassende **Insights** über das Unternehmensumfeld und dessen Veränderungen können mithilfe von Umfeld-Scanning-Software schnell und kontinuierlich erfasst werden. Dabei werden unter anderem relevante Trends für das jeweilige Unternehmen erfasst und genauer analysiert (z. B. künstliche Intelligenz für Prozessoptimierung oder virtuelle Agenten als neuer Service für Kundinnen und Kunden) und regelmäßige Leistungsvergleiche mit Wettbewerbern gemacht. Auf Grundlage dieser zuverlässigen Datenbasis fällt es leichter, fundierte Entscheidungen zu treffen, etwa welcher Modus – Exploit oder Explore – mit den verfügbaren Ressourcen und der jetzigen Wettbewerbslage geeigneter ist resp. welcher von diesen zwei Modi betont werden müsste. Wie diese beiden Modi zusammenspielen, zeigt das oben genannte Beispiel von Flixbus: Überprüfung neuer Dienste und strategische Entscheidungen auf Basis der Daten zum vergangenen Geschäft.

**Abb. 5:**
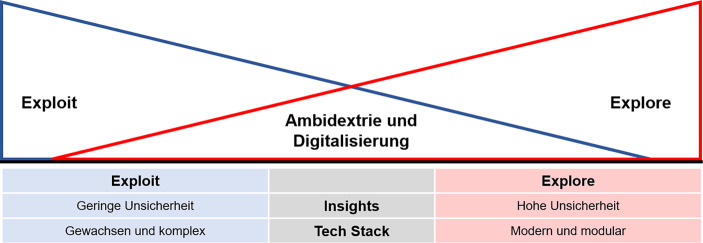
Ambidextrie und Digitalisierung – Exploit vs. Explore

Ist eine Entscheidung getroffen, zahlt ein moderner **Tech Stack** (also eine aktuelle und flexible IT-Infrastruktur) auf die notwendige Schnelligkeit und Flexibilität bei der Umsetzung ein. Abb. 5 fasst die wesentlichen Aspekte zusammen.

Im Exploit-Modus sind die Spielregeln bekannt: der Fokus liegt auf der Verbesserung bestehender Produkte, Dienstleistungen und Geschäftsmodelle für existierende Kundinnen und Kunden und Märkte, um die Kundenbindung und die Margen zu erhöhen. Die bekannten Wettbewerber werden intensiv analysiert, um das Differenzierungspotenzial zu heben. Unternehmen sammeln Daten von ihren Kundinnen und Kunden (mit ihrer Zustimmung), deren Einkaufsverhalten, oder führen Kundenbefragungen durch, um sich ein Bild weiterer Optimierungspotenziale zu machen. Die Datenbasis für **Insights** ist weitestgehend bekannt.

Im Explore-Modus verlassen Unternehmen die ihnen bekannte Welt. Nach Ansoff (1957) gibt es grundsätzlich drei Wachstumsstrategien für diesen Modus:

1. **Marktentwicklung:** Mit existierenden Angeboten neue Märkte erschließen
2. **Produktentwicklung:** Mit neuen Angeboten bekannte Märkte adressieren
3. **Diversifikation:** Mit neuen Angeboten neue Märkte erschließen

Je weiter sich ein Unternehmen vom Kerngeschäft entfernt, umso höher ist die Unsicherheit bezüglich eines erfolgreichen Product-Market-Fit. Entscheidungen unter extremer Unsicherheit können durch eine sehr gute Datenlage erleichtert werden. Hierfür muss man über die Grenzen der herkömmlichen Marktforschung hinausgehen und Daten aus etlichen verschiedenen Quellen sammeln und unter zahlreichen Gesichtspunkten interpretieren (Wiser et al. 2019). Dabei ist es wichtig, kontroverse Sichtweisen zuzulassen und verschiedene Expertinnen und Experten einzubeziehen. Es gibt eine Reihe an Methoden aus dem Strategic Foresight, Umfeld-Scanning und Innovationsmanagement, die erst handhabbar werden, wenn sie mit IT unterstützt werden. Die Szenariotechnik ist eine Methode, um in sich schlüssige alternative Zukunftsbilder zu entwerfen anhand derer verschiedene Strategien geprüft werden. Zum Beispiel entwickelte der Bund für Umwelt und Naturschutz zusammen mit der IG Metall Szenarien für den „Mobilitätssektor 2030“. Auf Basis der entstandenen alternativen Zukunftsbilder wurden strategische Handlungsoptionen für eine zukunftsorientierte Gestaltung des Mobilitätssektors entwickelt (BUND und IG Metall 2022). Für eine robuste Szenarioanalyse müssen etliche Faktoren gesammelt, Schlüsselfaktoren identifiziert und anschließend durch Expertinnen und Experten bewertet werden. Konsistenzanalysen und die anschließende Kalkulation der Szenarien würden manuell mehrere Monate dauern (Durst et al. 2015). Diese aufwändige Technik wird aktuell von großen Konzernen oder Instituten genutzt. Insbesondere KMUs sehen sich hier einem eindeutigen Wettbewerbsnachteil gegenüber Konzernen ausgesetzt. Im äußersten Fall führen sie diese Aktivitäten aufgrund der hohen Komplexität und den mit ihnen verbundenen Kosten nur unzureichend oder gar nicht durch. Das birgt die Gefahr, dass diese Unternehmen komplette Trends oder technologische Entwicklungen übersehen und infolgedessen innerhalb kürzester Zeit durch besser vorbereitete Wettbewerber oder Startups verdrängt werden. Mithilfe von Big Data Analytics können große Datenmengen schneller verarbeitet und analysiert werden. Methoden der Künstlichen Intelligenz helfen dabei, relevante Trends und Technologien schnell zu identifizieren (Kölbl et al. 2019). Unternehmen, die in der Lage sind, Informationen aus verschiedenen Datenquellen schnell zu kombinieren und zu interpretieren, können auch unter größerer Unsicherheit bessere Entscheidungen treffen und so schneller und zielgerichteter als der Wettbewerb agieren.

Wenn Entscheidungen gefallen sind und die Innovations-Roadmap steht, zählt Geschwindigkeit. Biontech hat sehr schnell den ersten COVID-19-Impfstoff entwickelt, va-Q-tec hatte genauso schnell die passenden Kühlboxen für den Transport und auch andere Unternehmen konnten sich in der Krise schneller als andere an neue Gegebenheiten anpassen. Während manche Unternehmen von jetzt auf gleich auf Telearbeit umstellen konnten, scheiterte es bei vielen an der nicht vorrätigen Hardware für die Mitarbeitenden oder der entsprechenden Infrastruktur. Ähnlich verhält es sich mit der Umsetzung neuer Produkte, Dienstleistungen oder Geschäftsmodelle. Erst, wenn ein Unternehmen vom Exploit-Modus in den Explore-Modus wechselt, zeigt sich, inwiefern der Tech Stack neuen Anforderungen gerecht wird, und die neuen Vorhaben beschleunigt, anstatt sie auszubremsen. In Zeiten, wo digitale Komponenten an Produkten oder Dienstleistungen zum Standard werden, oder neue Geschäftsmodelle ermöglichen, werden veraltete IT-Infrastrukturen zunehmend zum Problem. Große, etablierte Unternehmen mit starkem Fokus auf das Kerngeschäft verzeichnen häufig eine gewachsene und komplexe IT-Architektur mit hunderten oder tausenden IT-Anwendungen, die mehr oder minder gekoppelt ineinandergreifen. Die Modernisierung ist aufwändig und wird nicht selten hintenangestellt („*never touch a running system*“). Die damit entstehenden sogenannten Tech Debts werden im Zeitverlauf immer größer und erschweren die Umsetzung und Integration neuer Produkte und Dienstleistungen – also den Explore-Modus. Ein Großteil der IT-Teams ist damit beschäftigt, Komplexität zu verwalten, anstatt die IT vorausschauend auf die Zukunft auszurichten. Zudem verhindern komplexe und unabhängige Datenarchitekturen in Silos Unternehmen daran, ihre Daten vollumfänglich und in Echtzeit auswerten zu können und zu einer besseren Entscheidungsfindung einzusetzen. Oftmals ist es einfacher, neue explorative Ideen in andere Unternehmen oder Neugründungen auszugliedern, um nicht von einem Legacy-IT-Stack sowie gewachsenen Organisationsstrukturen ausgebremst zu werden. Der digitale Zustellservice pakadoo wurde 2015 als unternehmensinternes Startup der LGI Logistics Group International GmbH gegründet und adressiert mit seinen Services das Last-Mile-Problem. Im Dezember 2018 wurde pakadoo in eine eigenständige Gesellschaft umgewandelt, um losgelöst von etablierten Strukturen des Mutterkonzerns das neue Geschäftsmodell noch schneller weiterentwickeln zu können (LGI 2016 und 2018).

## Fazit

Ambidextrie steht sinnbildlich für die Fähigkeit, beide Hände gleich geschickt einzusetzen, und wird zunehmend im Unternehmensumfeld als Metapher für deren Flexibilität und Reaktionsfähigkeit verwendet. Im Innovationsmanagement bezieht sich Ambidextrie auf die Fähigkeit von Unternehmen, sowohl exploitative als auch explorative Aktivitäten durchzuführen, um sich an unsichere und sich verändernde Umweltfaktoren anzupassen. Exploitative Arbeiten beziehen sich eher auf inkrementelle Innovationen, die darauf ausgelegt sind, das Kerngeschäft zu stärken, während sich explorative Aktivitäten auf disruptive Innovationen fokussieren, um neue Lösungen auf den Markt zu bringen oder sogar in einen neuen Markt einzutreten. Aber weder der eine Modus noch der andere Modus ist langfristig zielführend. Um im Markt zu bestehen, gilt es die Fähigkeit zu erlangen, mit beiden Modi agieren zu können – die Fähigkeit, mit den gegebenen Ressourcen und der aktuellen Wettbewerbslage, entweder mehr in den Exploit- oder mehr in den Explore-Modus zu investieren. Diese Fähigkeit, einerseits beide Modi zu leben und andererseits beide Modi in einer Art und Weise miteinander zu verflechten, stellt aber sowohl für traditionelle Unternehmen, die eher im Exploit-Modus agieren, als auch für Start-ups, die wiederum eher explorativ unterwegs sind, eine Herausforderung dar.

Abhängig davon, wie die Unternehmensstrategie ausgelegt ist, wie die Strukturen und Prozesse innerhalb eines Unternehmens aufgebaut und gelebt sind, welches Führungsverständnis herrscht, welche Kultur gelebt werden kann und wie mit Wissen und Potenziale im Unternehmen umgegangen wird, ist das Unternehmen entweder eher im Exploit- oder im Explore-Modus unterwegs. Wie zu Beginn des Beitrags erwähnt, müssen heutige Unternehmen weg vom „entweder-oder“, sondern mehr in Richtung „sowohl-als-auch“ gelangen. Ein ambidexteres Innovationsmanagement bietet Unternehmen zahlreiche Vorteile. Beherrscht ein Unternehmen beide Innovationsmodi, kulturell wie auch im Führungshandeln, und kann die Modi gezielt für das Unternehmen einsetzen, so können menschliche Barrieren oder Fehleinschätzungen überwunden werden. Die Fähigkeit der Beidhändigkeit ermöglicht einerseits, sich durch Optimierungspotenziale in gesättigten Märkten zu behaupten und andererseits neue Chancen in Form von innovativen Produkten, Dienstleistungen und Geschäftsmodellen in bestehenden oder neuen Märkten zu erschließen. Damit dies besser gelingt, helfen digitale Lösungen, zum Beispiel datenbasiert Insights zu erfassen und diese mithilfe von Big-Data-Analytics-Tools und künstlicher Intelligenz zu analysieren, um daraus Entscheidungen abzuleiten.

Führungskräfte sind gefordert, in allen Situationen klar zu sehen, wie sie zu bewerten und ihnen zu begegnen sind. Dabei verlangen die heutigen und sehr wahrscheinlich auch die zukünftigen Märkte gut balancierte Lösungen zur richtigen Zeit. Da hilft weder der bedachte noch der mutige Typ, sondern nur ein Mix von beiden. Dies erfordert neben der Ambidextrie im Innovationsmanagement auch eine gewisse Ambiquitätstoleranz, die Fähigkeit, mit komplexen Welten, die unklar, unsicher und punktuell widersprüchlich sind, umgehen zu können. Ein Werkzeugkoffer beider Innovationsmodi hilft, die jeweils passende Ausprägung vom Exploit oder Explore zu wählen und diese gezielt einzusetzen. Dadurch erhöht sich die Reaktions- und Anpassungsfähigkeit auf neue Gegebenheiten, sei es etwa sich neu zu organisieren und bestehende Prozesse zeitnah den gegebenen Umweltfaktoren anzupassen, wie es etwa die Covid19-Pandemie von uns verlangte. Dies bedingt jedoch, dass auf allen Ebenen, von der Führungskraft bis zum einzelnen Mitarbeitenden, die Ambidextrie verstanden und verinnerlicht ist.

Es ist wichtig, das Potenzial von Ambidextrie im eigenen Unternehmen zu verstehen und zu wissen, wann und wie welcher Modus – Exploit oder Explore – für das Unternehmen von Nutzen ist. Dies kann sich positiv auf verschiedene Aspekte wie die Effizienz, den Gewinn und die Unternehmenskultur auswirken. Aber auch auf die Mitarbeitenden selbst. Eine ambidextere Kultur ermöglicht eine bessere Lern- und Fehlerkultur, da Mitarbeitende ermutigt werden, gewohnte Pfade zu verlassen und eigenverantwortlich neue Wege zu gehen. Dies führt langfristig zu einer Erhöhung der Innovationskraft und ermöglicht die Entstehung neuer Ideen.

Nicht zuletzt zahlt eine ambidextre Struktur auf die Attraktivität des Unternehmens als Arbeitgeber ein. Eine selbstführungsfördernde Kultur, ein sinnstiftendes Arbeitsumfeld und eine Fehlerkultur werden von vielen jungen Talenten als wichtigste Faktoren bei der Wahl eines Arbeitsplatzes betrachtet. Unternehmen, die eine solche Kultur fördern, positionieren sich so als attraktive Arbeitgeber und gewinnen Talente, die das Unternehmen weiter vorantreiben können. Insgesamt bietet die Ambidextrie somit eine vielversprechende Möglichkeit, Unternehmen langfristig zu stärken und auf Veränderungen schnell und erfolgreich reagieren zu können.

Es zeigt sich, dass mit oder trotz der Digitalisierung, welche für die steigende Nutzung und Akzeptanz von digitalen Lösungen steht, die Fähigkeit der Nutzung beider Hände, der traditionellen Hand in Kombination mit der neu-denkenden Hand (oder umgekehrt), notwendig ist, um weiterhin im Markt zu bestehen oder einen neuen Markt einzutreten. Dabei ist weder der Exploit- noch der Explore-Modus „richtig“, sondern je nach Unternehmen und Branche gibt es eine eigene Zusammenstellung, die für das jeweilige Unternehmen optimal ist. Ambidextrie gilt dabei besonders in schlechteren Zeiten als Schlüsselfaktor.

In diesem Sinne: „*You cannot overtake 15 cars in sunny weather, but you can when it’s raining*“ (Zitat von Ayrton Senna, brasilianischer Formel 1 Fahrer). Es ist also ratsam, bereits in guten Zeiten eine Ambidextrie im Unternehmen zu fördern und beide Modi im Sinne des Unternehmens einzusetzen, um für die Zukunft gewappnet zu sein.
